# Follow-Up for an Abnormal Newborn Screen for Severe Combined Immunodeficiencies (NBS SCID): A Clinical Immunology Society (CIS) Survey of Current Practices

**DOI:** 10.3390/ijns6030052

**Published:** 2020-06-30

**Authors:** Vijaya Knight, Jennifer R. Heimall, Nicola Wright, Cullen M. Dutmer, Thomas G. Boyce, Troy R. Torgerson, Roshini S. Abraham

**Affiliations:** 1 Division of Allergy and Immunology, Department of Pediatrics, University of Colorado School of Medicine, Children’s Hospital Colorado, Aurora, CO 80045, USA; Cullen.dutmer@childrenscolorado.org; 2 Division of Allergy and Immunology, Children’s Hospital of Philadelphia, Perlman School of Medicine at University of Pennsylvania, Philadelphia, PA 19104, USA; heimallj@email.chop.edu; 3 Department of Pediatrics, Alberta Children’s Hospital, University of Calgary, Calgary, AB T3B 6A8, Canada; Nicola.Wright@albertahealthservices.ca; 4 Division of Pediatric Infectious Diseases, Marshfield Clinic, WI 54449, USA; boyce.thomas@marshfieldclinic.org; 5 Experimental Immunology, Allen Institute, Seattle, WA 98109, USA; troy.torgerson@alleninstitute.org; 6 Department of Pathology and Laboratory Medicine, Nationwide Children’s Hospital, Columbus, OH 43205, USA; Roshini.Abraham@nationwidechildrens.org

**Keywords:** severe combined immunodeficiencies, TREC, newborn screening, lymphocyte quantitation, flow cytometry, naïve and memory T cells

## Abstract

Severe combined immunodeficiency (SCID) includes a group of monogenic disorders presenting with severe T cell lymphopenia (TCL) and high mortality, if untreated. The newborn screen (NBS) for SCID, included in the recommended universal screening panel (RUSP), has been widely adopted across the US and in many other countries. However, there is a lack of consensus regarding follow-up testing to confirm an abnormal result. The Clinical Immunology Society (CIS) membership was surveyed for confirmatory testing practices for an abnormal NBS SCID result, which included consideration of gestational age and birth weight, as well as flow cytometry panels. Considerable variability was observed in follow-up practices for an abnormal NBS SCID with 49% confirming by flow cytometry, 39% repeating TREC analysis, and the remainder either taking prematurity into consideration for subsequent testing or proceeding directly to genetic analysis. More than 50% of respondents did not take prematurity into consideration when determining follow-up. Confirmation of abnormal NBS SCID in premature infants continues to be challenging and is handled variably across centers, with some choosing to repeat NBS SCID testing until normal or until the infant reaches an adjusted gestational age of 37 weeks. A substantial proportion of respondents included naïve and memory T cell analysis with T, B, and NK lymphocyte subset quantitation in the initial confirmatory panel. These results have the potential to influence the diagnosis and management of an infant with TCL as illustrated by the clinical cases presented herein. Our data indicate that there is clearly a strong need for harmonization of follow-up testing for an abnormal NBS SCID result.

## 1. Introduction

Severe combined immunodeficiency (SCID) includes a group of monogenic disorders, which are characterized by significant T cell lymphopenia (TCL) in the neonate, present either in isolation or with diminished counts in other lymphocyte subsets, including B and Natural Killer (NK) cells. There are at least 20 different genetic defects associated with SCID (1) with additional genetic defects that may be associated with a SCID-like phenotype [1,2]. SCID was added to the recommended universal screening panel (RUSP) for newborn screening in 2010 in the United States. Pilot screening was initiated in 2008 [3] and by the end of 2018, all 50 states within the US and several other countries had adopted the use of the T cell receptor excision circle (TREC) biomarker for the newborn screen for SCID (NBS SCID) [4,5,6,7,8]. Although SCID is the primary target of newborn screening, widespread implementation revealed that other conditions with TCL are also identified, including some syndromic disorders with early-onset TCL such as 22q11 deletion syndrome, secondary causes of TCL, and prematurity [9,10,11,12,13,14,15]. In addition, while TREC quantification has proved to be an effective biomarker for most cases of SCID, there is concern that some defects have a higher potential to be missed, in particular those due to ADA deficiency for which mass spectrometry has been used [16]. While TREC quantification is useful in identification of early onset TCL, it is not effective in identifying late onset TCL (e.g., Bare Lymphocyte Syndromes due to MHC class I or II deficiencies, DOCK8 deficiency, ZAP70 deficiency among others) [17,18].

There is no universally accepted process for evaluating an abnormal NBS SCID result. Most screening laboratories may repeat testing internally, refer the newborn for clinical follow-up and additional flow cytometry analysis, or may utilize specific confirmatory approaches for premature or low birth weight (LBW) infants. This diversity in follow-up has precluded a facile harmonization of post-screen abnormal results.

**Figure 1:**
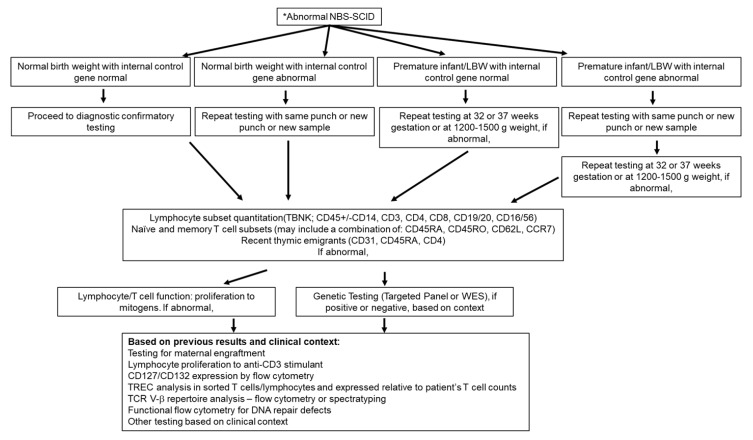
Follow up testing strategies for an abnormal newborn screen for severe combined immunodeficiency (*NBS SCID) result. NBS SCID includes PCR amplification of T cell receptor excision circle (TREC) and an internal control gene, generally beta-actin or RNAse P. An abnormal result (low TREC and/or low internal control gene) may trigger confirmatory flow cytometry testing, repeat DBS and/or repeat TREC analysis or repeating DBS and TREC analysis in preterm infants when closer to term birth weight or age. Flow cytometry analysis includes basic lymphocyte subset phenotyping and may include additional markers for naïve and memory T cells and recent thymic emigrants, followed by analysis of T cell function, genetic and other immunological analyses as deemed appropriate.

Once an NBS SCID result has been conclusively established as being abnormal, further follow-up typically occurs outside the public health screening laboratory, with confirmation being performed in a clinical diagnostic laboratory. This confirmatory testing is essential to pursuing a definitive diagnosis and subsequent management strategies. The most widely used first tier of confirmatory testing after a complete blood count with differential (CBC) is quantitation of lymphocyte subsets (total lymphocytes (CD45+ absolute lymphocyte count), CD3+, CD4+, and CD8+ T cells; CD19+ or CD20+ B cells; and CD3-/CD16+ and/or CD56+ NK cells) by flow cytometry. This method rapidly allows confirmation of severe TCL, while also revealing any deficits in other lymphocyte subsets and allows for triage of the common genetic defects. However, there is heterogeneity at this level of evaluation and confirmation as well, with some laboratories and centers including evaluation of naïve and memory T cells and/or recent thymic emigrants (RTEs) by flow cytometry, and others reserving this analysis for a subsequent stage of confirmation, based on T, B, and NK quantitation results. Yet some directly proceed from these immunophenotyping analyses to functional studies, with measurement of T cell function by lymphocyte proliferation to mitogens, such as phytohemagglutinin (PHA), while others may pursue such testing in a sequential manner. Additional immunological or genetic investigations are typically reserved for later evaluation, based on the result of preceding laboratory data (Figure 1).

The Primary Immunodeficiency Treatment Consortium (PIDTC) has developed diagnostic criteria for typical and leaky SCID using analysis of lymphocyte subsets including naïve and memory T cells along with T cell proliferation to PHA [19] (Table 1). These analyses are also of utility in assessing other causes of TCL identified by NBS SCID such as variant SCID or idiopathic TCL, syndromes associated with TCL, secondary TCL, and prematurity (Table 1) [20]. 

To investigate the diversity of approaches used to further evaluate an abnormal NBS SCID result, the Clinical Immunology Society (CIS), conducted a survey of its membership who are largely but not exclusively immunologists. The goal of the survey was to collect information on current practices utilized to evaluate infants with an abnormal TREC-based NBS SCID result and provide recommendations for optimal laboratory follow-up.

## 2. Methods

The survey was administered to the CIS membership between November and December 2018 (Table 2). For assessment of NBS SCID practices, the survey included questions to evaluate (a) whether centers utilized a one-step (an abnormal NBS SCID proceeds to confirmatory flow cytometry testing), a two-step (an abnormal NBS SCID proceeds to repeat TREC analysis), or alternative algorithms, and (b) gestational age and birth weight considerations when evaluating the results of a NBS SCID. Regarding follow-up testing for an abnormal NBS SCID, the survey questions were intended to assess (a) the scope of flow cytometry assays that are currently used to confirm an abnormal NBS SCID, and (b) whether centers followed a tiered approach to flow cytometry and functional immunological testing. Respondents were also requested to provide additional comments related to these questions, if so desired. 

The survey was sent to all members of the CIS (approximately 900+) and data were collected and analyzed. 

## 3. Results

### 3.1. Survey Data

**Figure 2:**
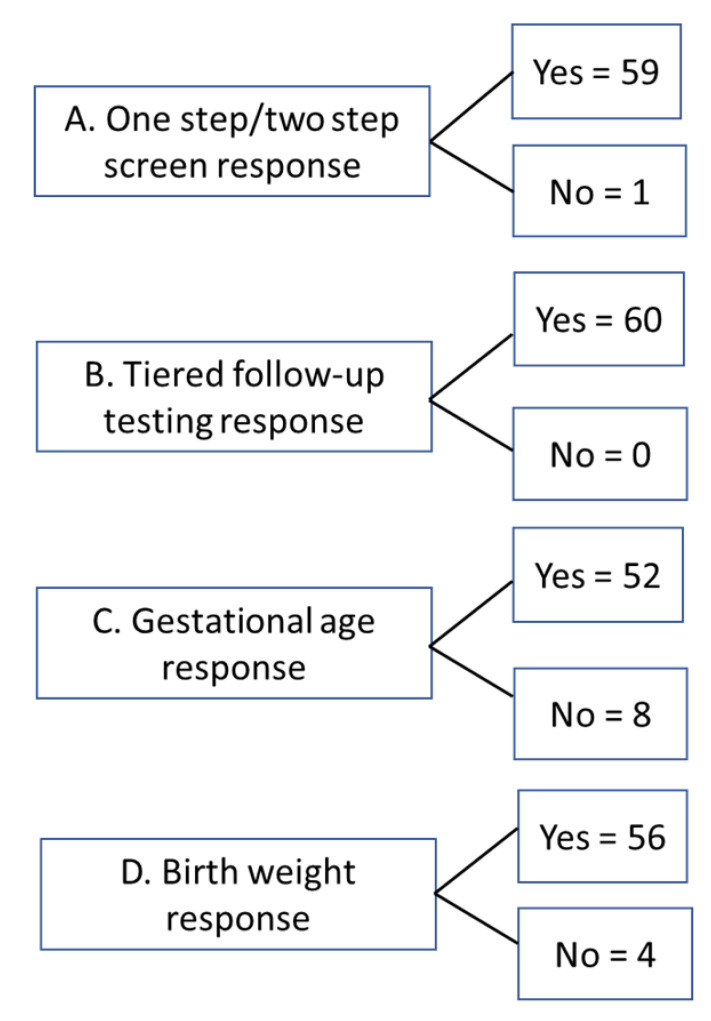
Response rate for survey questions. Overall, sixty responses were received to the survey. Not all questions were answered by all respondents.

A total of 60 surveys were completed, representing approximately 7% of CIS membership. Not all respondents answered all four survey questions. We attempted to collect additional data from the unanswered questions from the respondents. The breakdown of responses obtained for each of the 4 questions is shown in Figure 2.

Respondents were from the United States, Canada, Brazil, New Zealand, Turkey, Egypt, Spain, and Lebanon. All data were included in the analysis regardless of whether respondents had access to a formal national- or state-run NBS SCID program or not. 

### 3.2. Follow-Up to an Initial Abnormal NBS SCID

Our survey indicated that the majority of respondents (29/59 or 49%) utilized a one-step NBS SCID screening protocol, indicating that an abnormal NBS SCID result was referred for confirmation by flow cytometry analysis. Twenty-three respondents (39%) followed a two-step protocol whereby an abnormal NBS SCID was automatically reflexed to repeat TREC analysis to confirm or negate the result prior to referral for flow cytometry testing. Seven respondents chose “other” in response to this question. Further correspondence with these respondents revealed that their centers utilize birth weight or gestational age as additional criteria to either refer an abnormal NBS SCID result for additional flow cytometry analysis, repeat TREC analysis, or for repeat dried blood spot (DBS) collection and TREC analysis in a LBW or pre-term infant once closer to term. 

Interestingly, our survey data indicate that only a minority of respondents took birth weight and age into account when following up on an abnormal NBS SCID result. 

Gestational age was not included as criteria by 53% of respondents. Of the remaining respondents, 30% indicated ≥35 weeks of gestation as their cut-off for their specific follow-up protocol for an abnormal NBS SCID result, 13% indicated 32–34 weeks, and 4% indicated less than 30 weeks. Similarly, the majority of respondents indicated that birth weight, as a parameter was not applicable when evaluating abnormal NBS SCID results. Of the 56 respondents, 43 or 77% indicated that weight was not considered in the follow up of an abnormal NBS SCID test result. The cut-off values for weight varied from 500–2800 g, with relatively few respondents including birth weight as a decision-making parameter for confirming an NBS SCID result.

### 3.3. Confirmatory Testing

Once the TREC-based screen is referred for follow-up outside of the public health lab, confirmation of an abnormal NBS SCID is typically performed by flow cytometry evaluation of a peripheral blood sample for T cell subsets, B cells, and NK cells. The addition of CD45RA (naïve T cells) and CD45RO (memory T cells) markers is increasingly being incorporated into confirmatory flow cytometry testing protocols for early detection of maternal engraftment of T cells or leaky SCID phenotypes. A T cell proliferative response to PHA has also been incorporated by some laboratories into their confirmatory panels for an abnormal NBS SCID [21].

Our survey results indicated that there were five typical approaches to confirmatory testing: The most common initial approach, employed by 47% of respondents, utilized T, B, and NK lymphocyte subset and naïve and memory T cell quantitation as the initial follow up testing for an abnormal NBS SCID, followed by T cell proliferation to PHA. The second most common approach, employed by 22% of respondents, utilized T, B, and NK lymphocyte subset and naïve and memory T cells quantitation with T cell proliferation to PHA in the first step of confirmation. A minority (15%) utilized T, B, and NK lymphocyte subset quantitation alone for confirmation, followed by naïve and memory T cell analysis and PHA response in a second step, and even fewer (6%) utilized T, B, and NK lymphocyte subset quantitation for confirmation followed by analysis of naïve and memory T cell populations with no assessment of T cell proliferation. A response of “other” was selected by 10%. Correspondence with these respondents indicated that two abnormal NBS SCID results followed by abnormal lymphocyte subset quantitation (T, B and NK) immediately prompted genetic testing. Respondents from reference laboratories indicated that additional flow cytometry analysis, such as assessment of naïve and memory T cells or T cell proliferation was only performed at the request of the ordering physician. 

### 3.4. Utility of Naïve, Memory, and RTE Phenotyping in Addition to T, B, and NK Analysis

The case examples below highlight the advantage of including naïve and memory T cell- and RTE markers in the early laboratory assessment of an abnormal NBS SCID result.

Case 1. A male infant had an abnormal NBS SCID, performed at the Minnesota State Health Laboratory under the NBS screening program, and was referred for further evaluation. The original newborn screening TREC result was not available, however, analysis performed on peripheral blood mononuclear cells (PBMC) confirmed the abnormal result (TREC = 1158 copies/10^6^ CD3^+^ T cells (reference range >4168 copies/10^6^/ CD3^+^ T cells)). The patient’s clinical history was remarkable for initial failure to thrive (FTT) but with subsequent normal growth and development. The remainder of the patient’s clinical history was unremarkable. The lymphocyte subset quantitation revealed a T^low^B^low^NK^normal^ immunophenotype (Table 3).

**Figure 3:**
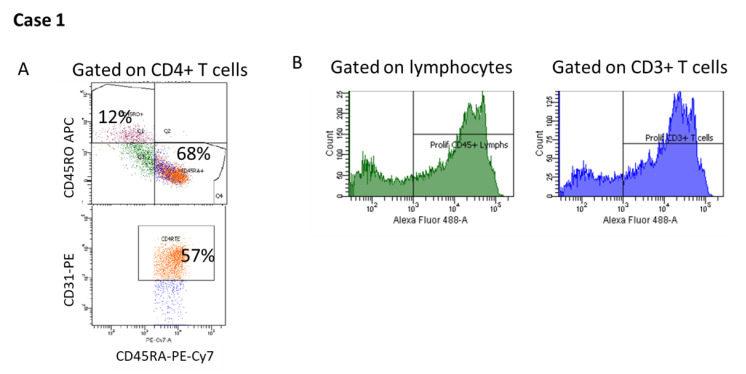
Case 1, phenotype and functional assessment of lymphocyte populations. (**A**) Relative percentages of naïve (CD45RA+) and memory (CD45RO+) T cells, and recent thymic emigrant (CD45RA+CD31+) T cells; (**B**) Proliferation of CD45+ lymphocytes and CD3+ T cells in response to phytohemagglutinin (PHA) stimulation. Peripheral blood mononuclear cells were stimulated with PHA for 72 h. The cells were incubated with a modified nucleoside (5-Ethynyl-2′-deoxyuridine) and then labeled with a fluorescent dye. Proliferating cells (CD45+ lymphocytes and CD3+ T cells) were identified by an increase in fluorescence.

The significant TCL with modest B cell lymphopenia warranted further investigation of the T cell compartment. Flow cytometric analysis of naïve and memory T cells and RTEs, performed at one week of age revealed that naïve CD45RA+CD4+ T cells and memory CD45RO+CD4+ T cells were within normal limits for age and RTEs were modestly decreased (Figure 3A). TREC analysis of CD3+ T cells indicated that there was impaired but not completely absent thymic function. This result was not consistent with typical or leaky SCID. T cell stimulation with PHA revealed normal total CD45+ lymphocyte and CD3+ T cell proliferation (Figure 3B). FISH analysis for a deletion at position 22q11.2 was positive, revealing a diagnosis consistent with partial DiGeorge Syndrome (DGS), even though the patient did not have other characteristic features associated with this condition. Although the initial lymphocyte subset quantitation revealed a substantial TCL, the patient possessed naïve T cells and RTE, with normal T cell proliferation to PHA, all of which are inconsistent with typical SCID [21].

**Figure 4:**
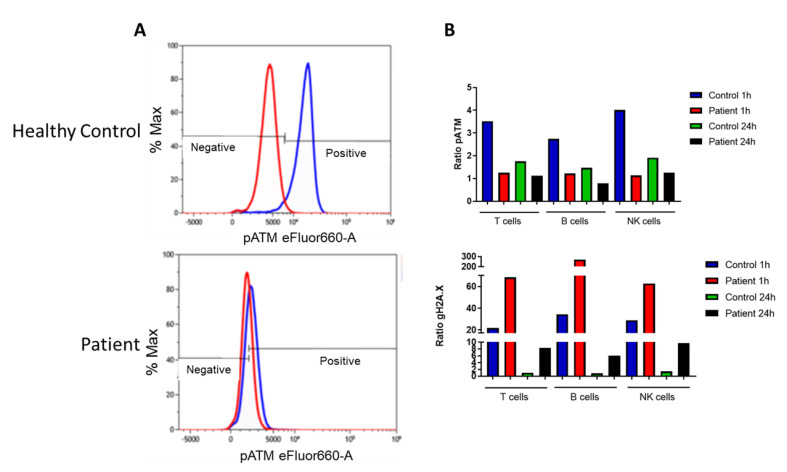
Case 2, Analysis of pATM and gamma H2AX. (**A**) Analysis of pATM in CD3+ T cells in healthy control and patient T cells. Red histograms represent non-irradiated cells; blue histograms represent cells exposed to low dose (2 Gy) radiation. The phosphorylated proteins were assessed at 1 h and 24 h post-irradiation. (similar data was obtained for patient B and NK cells (data shown in bar graph, (**B**)). (**B**) The median mean fluorescence intensity (MFI) was plotted as a ratio of irradiated to unirradiated data for pATM (top graph) and gamma H2AX (bottom graph). The patient (red bars) shows almost absent (close to a ratio of 1) ATM phosphorylation compared to an experimental healthy control (blue bar) at 1 h post-2Gy irradiation. At 24 h post-irradiation, the control (green bar) shows normal DNA repair and dephosphorylation of pATM, while the patient (black bar) shows no change from the 1 h time point for T, B, and NK cells. In the lower graph, the median MFI ratio for gamma H2AX is depicted, which shows significantly higher expression in the patient (red bar) compared to control (blue bar) at 1 h post-irradiation, suggesting increased foci of DNA DSB. At 24 h post-irradiation, the patient (black bar) shows persistence of gamma H2AX compared to the control (green bar), indicating defective DNA repair.

Case 2. A female infant had an abnormal NBS SCID, performed at the North Carolina State Health Laboratory. The abnormal result was confirmed with repeat NBS SCID and the infant was referred for further evaluation. TREC values from the newborn screen were unavailable. Flow cytometric analysis revealed T and B cell lymphopenia (Table 4). Her serum IgG was 645 g/dL but likely of maternal origin, and her IgM and IgA were below the limit of detection. Due to concern for SCID or leaky SCID, additional flow cytometric studies to evaluate naïve and memory T cells and RTEs were performed (Table 4). The results were not consistent with SCID, despite lower than normal naïve T cells. An initial SCID genetic panel revealed a heterozygous deletion of the entire coding region of *CORO1A* (encoding the coronin1-A protein, CORO1A). As an autosomal recessive condition, CORO1*A* deficiency is usually associated with a syndromic SCID phenotype in the biallelic state. Additional genetic testing was recommended, which revealed compound heterozygous variants in trans in *ATM* (encoding ataxia telangiectasia mutated, ATM), consistent with a diagnosis of ataxia telangiectasia (AT), a syndrome associated with neurological defects, immunodeficiency, and cancer predisposition. Functional flow cytometry to characterize the *ATM* variants revealed absent ATM phosphorylation on induction of DNA double-strand breaks (DSBs) via low-dose (2Gy) radiation to lymphocytes (Figure 4A). Interestingly, gamma H2AX, an early marker of DNA DSB repair was significantly increased at both 1 h and 24 h post-radiation (Figure 4B). Gamma H2AX is normally increased in contexts of increased foci of DNA DSB and impaired DNA repair. However, in most cases of AT, gamma H2AX, which is phosphorylated by ATM and ATR (ATM-and Rad3-related kinase), is half that of normal due to the lack of functional ATM. In this patient the increased gamma H2AX at 1h, and persistence at 24 h post-radiation, suggested multiple foci of DNA DSBs, which had not been repaired normally. 

Case 3: A male infant had an abnormal NBS SCID with undetectable TRECs, performed at two days of age. As per the New Mexico State Laboratory NBS SCID protocol, a repeat NBS SCID, performed at 10 days of age, confirmed undetectable TREC and the infant was referred for further evaluation. Initial flow cytometric analyses revealed profound TCL and normal B cell and NK cell counts, suggesting a T^low^B^normal^NK^normal^ immunophenotype (Table 5). The patient had no anatomical anomalies or facial dysmorphisms. An initial SCID genetic testing panel revealed a heterozygous variant in *JAK3*. Biallelic mutations in *JAK3* result in JAK3 deficiency, which is an autosomal recessive SCID condition that typically manifests with a T^low^B^normal^NK^low^ immunophenotype, and therefore neither the immunophenotype of the patient nor the identified heterozygous variant adequately explained his clinical phenotype. At seven weeks of age, his T cell count rose to 1100 cells/µL with a normal CD4:CD8 ratio. However, naïve and memory T cell analyses revealed that nearly all of his CD4+ T cells were CD45RO+. Engraftment of maternal T cells was not detected, while TCR-Vβ spectratyping on the T cells of the patient revealed oligoclonality. The increased T cell count was accompanied by widespread dermatitis, erythroderma, and skin desquamation akin to Omenn syndrome, for which he was ultimately treated with Alemtuzumab. Since whole genome sequencing with copy number variation analysis and a chromosomal microarray did not identify a genetic etiology of his apparent T cell defect, CD34+CD3- hematopoietic stem and progenitor cells were enriched from the bone marrow of the patient and cultured in an artificial thymic organoid environment as previously described by Seet et al. [22], which revealed grossly normal T cell maturation, suggesting that HCT may not be an optimal or curative treatment for this patient. Therefore, he is awaiting allogeneic thymus transplantation. 

Case 4: A female infant was referred for further evaluation following a presumptive abnormal NBS SCID result from the Pennsylvania newborn screening laboratory. TREC values from the newborn screen were unavailable, however, TREC analysis performed on PBMC at 13 days of age revealed low TRECs (846 copies/10^6^ CD3+ T cells), thus confirming the initial abnormal result. The patient had no anatomical anomalies or facial dysmorphisms. Initial flow cytometry results, inclusive of naïve and memory T cells are presented in Table 6. Lymphocyte proliferation to PHA was mildly reduced (41% of normal). Taken together, these laboratory findings were concerning for leaky SCID with a T^low^B^low^NK^normal^ phenotype. Maternal engraftment was not detected. Although her overall white blood cell count was normal, the differential was notable for elevated eosinophils. An SCID genetic testing panel revealed two deleterious variants (compound heterozygous) in *RAG1* (heterozygous p.W204X, p.W522C). She was then successfully treated with HCT.

## 4. Discussion

**Figure 5:**
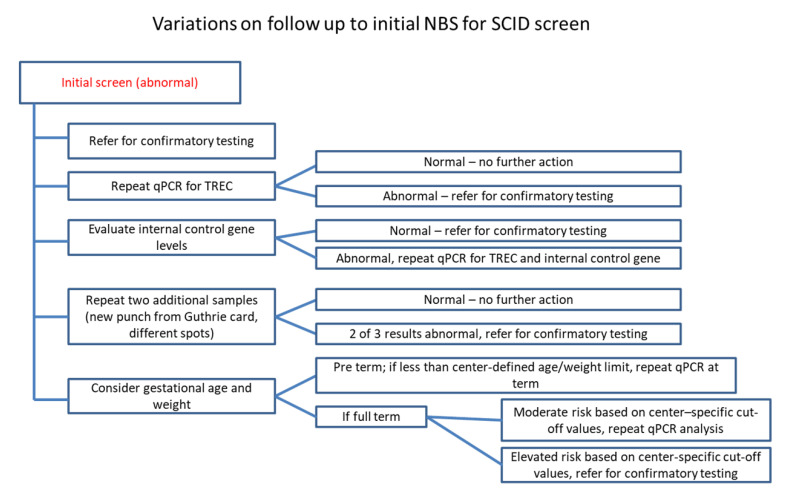
Variations on follow up practices for an abnormal NBS SCID result. Based on our survey results, respondents followed a diverse set of approaches to follow up on an abnormal NBS SCID result.

Since its development in 2005 [24], TREC quantitation has been accepted as a surrogate measure of T cell output from the thymus. It is now widely implemented across the United States as a screening tool for SCID and related disorders with significant TCL [24]. Early diagnosis of SCID has led to expedited interventions and improved outcomes, including survival [25,26,27,28,29]. Despite successful implementation of the NBS SCID across the United States, there is currently little uniformity in screening and confirmatory testing strategies used by various state laboratories, tertiary centers, and consulting immunologists/providers. Usually, the initial NBS SCID includes quantitation of TRECs and an internal control gene (β-actin or RNaseP) using DNA extracted from a DBS obtained via a heel stick [30]. The actionable cut-off values for TREC quantitation vary widely, as do the algorithms for follow-up to an abnormal TREC screen [31]. An initial abnormal NBS SCID result may result in re-testing extracted DNA for confirmation of the abnormal result, or a peripheral blood sample may be collected for flow cytometry analysis of lymphocyte subsets, whereas failure of amplification of the control gene indicating questionable integrity of the extracted DNA may lead to repeat collection of a DBS and subsequent TREC quantitation. In concordance with published data, our survey data, though limited to 60 participants, indicated considerable heterogeneity in testing algorithms and are summarized in Figure 5. The variations noted in our survey data regarding follow-up to an initial abnormal NBS SCID result included referral for confirmatory testing by flow cytometry, repeat TREC analysis to confirm the initial result, evaluation of control gene levels to determine if the abnormal screen was due to amplification failure (inadequate or low quality DNA), repeat DNA extraction from different spots on the Guthrie card, or consideration of gestational age and/or birth weight prior to follow up testing for confirmation. 

Since the implementation of the NBS SCID, it has been noted that premature infants have a higher incidence of abnormal NBS SCID results compared with full-term infants (>37 weeks gestational age) [3,32,33,34]. Data from the Wisconsin SCID newborn screening program suggest that TREC values increase by 9.8% for every week of gestation [35]. Data from Spain demonstrates that TREC values are also lower in infants with LBW, with a positive correlation with gestational age [36]. These data and recently published reference ranges for term and preterm neonates without immune deficiency [37], are currently the only information available that define normal TREC levels or age-matched reference values for lymphocyte subsets in premature infants on a population level. The presence of other confounding factors associated with prematurity, including administration of prenatal corticosteroids for lung maturation, affects the interpretation of NBS SCID and confirmatory test results in these infants, leading to an increase in false positives. There are currently no definitive guidelines for follow-up to an abnormal NBS SCID result in premature or LBW neonates, and management of such infants is specific to the protocols established by an individual center or institution. However, there are efforts to address NBS SCID follow-up testing as a whole through the Clinical Laboratory Standards Institute guidelines document, which will be published in late 2021 [20]. Based on their experience of population-based newborn screening for SCID, Wisconsin and New York have implemented repeat NBS SCID testing in premature infants until the test is normal or until they reach an adjusted gestational age (AGA) of 37 weeks [38,39]. California adopted a second NBS SCID test to confirm or refute the original result regardless of gestational age. 

Although an abnormal NBS SCID result represents a false positive result in premature infants, it is important for practitioners to recognize that such infants may in fact have SCID. Additional precautions, such as initiation of strict isolation procedures and restriction of breastfeeding by CMV-positive mothers may be warranted until repeat TREC testing is normal or when the infant reaches an AGA of 37 weeks and an abnormal NBS SCID result can be confirmed. Low TREC results in premature infants may reflex to a second DBS and NBS SCID test, however, undetectable TREC, regardless of age, warrants immediate referral to an immunologist [39]. 

Although a CBC with differential can provide a quick and high-level assessment of lymphopenia in an infant, detailed evaluation of T, B, and NK lymphocytes is only possible via flow cytometry. This rapid and highly sensitive method provides both relative and absolute (cells/µL) quantitation using either a single or dual platform, depending on the laboratory and assay used. While quantification of CD4+ and CD8+ T cells (including total CD3+ T cells), B cells, and NK cells is readily available in most commercial labs and can detect severe TCL with or without associated defects in B and NK cells, this level of quantitation is limited by the inability to determine if the T cells present are of a naïve or memory phenotype. It is critical to determine if any T cells that are present are of a naïve or memory phenotype, since naïve T cells are indicative of thymic function, which is normally robust and the main source of T cells in an infant. In contrast, an expanded population of memory T cells can be seen in patients with either maternal engraftment of T cells or peripheral expansion of aberrant autologous T cells, as occurs in some hypomorphic forms of SCID. Therefore, incorporation of additional markers on CD4+ and CD8+ T cells, to identify naïve and memory subsets, is a critical component in the evaluation of neonatal TCL. Some of these additional surface markers can include CD45RA (a marker for naïve T cells), CD45RO (a marker for memory T cells), CD62L (a homing marker for naïve T cells), CCR7 (a chemokine receptor expressed primarily on naïve T cells), or CD31 (a marker for CD4+ RTEs). In neonates and infants, the vast majority of both CD4+ and CD8+ T cells, are naïve (CD45RA+CD62L+ or CCR7+) or “true” naïve (CD31+CD45RA+CD4+). In contrast, in the setting of maternal engraftment, Omenn Syndrome or leaky SCID there is typically an expansion of T cells with a memory phenotype in addition to a paucity of naïve T cells. When T cells are identified to be predominantly of a memory phenotype, maternal engraftment should be investigated by other methods, including fluorescence in situ hybridization (FISH) or short tandem repeat (STR) analysis. Infants with Omenn syndrome or leaky SCID typically demonstrate oligoclonal expansion of autologous T cells, which can be confirmed by TCR V-β repertoire analysis. In infants with a T^low^B^normal^NK^normal^ phenotype it is also important to ensure that the TCL is not due to a primary thymic defect (e.g., complete DiGeorge syndrome [21], FOXN1 deficiency [40], PAX1 defect [41] and Yamazaki Y et al., (manuscript submitted, 2019) which requires thymus transplantation as opposed to HCT.

Review of the Alberta, Canada cohort of SCID patients from 2009–2018 demonstrates the importance of inclusion of naïve and memory T cells in the evaluation of patients with possible SCID. Though the NBS SCID has only recently become available in Alberta, lymphocyte subset analysis has automatically included naïve CD4+ and CD8+ CD45RA+ T cell subsets during this period. Of 15 patients with a confirmed diagnosis of SCID, measurement of naïve T cells was instrumental in making the diagnosis of SCID in four patients (27%) due to normal to increased numbers of T cells and significantly low naïve T cell numbers in the context of Omenn syndrome (Table 7). 

T cell proliferation responses to PHA are generally incorporated into the algorithm for follow-up testing for an abnormal NBS SCID result. Since most of these infants have extreme TCL, the use of flow cytometry methodology to assess T cell function to mitogenic stimuli provides an analytical advantage and enhanced sensitivity over traditional radiometric assays. This is useful in distinguishing between infants with TCL who have normal versus abnormal T cell function [42].

The interpretation of all the above immunological testing, but particularly the quantitative immunophenotyping, is heavily dependent on having age-appropriate reference values for patient data comparison. Many laboratories struggle to find adequate numbers of healthy infants and children to develop a robust age-matched reference range. This challenge is even more obvious in premature infants; however, a recent study has alleviated this concern to some extent by publishing flow cytometric data from a relatively large cohort of premature infants and term neonates [37]. It is ideal for every reference laboratory to develop its own reference intervals as there can be assay and instrument-specific variability in results of lymphocyte immunophenotyping, and it is best to compare patient data with control data generated within the same laboratory. Nevertheless, this may not always be practical or feasible, and in such situations, published reference ranges may be utilized as an alternative.

Cases 1, 2, and 3 described here provide compelling arguments for including naïve and memory T cell markers in the early confirmatory evaluation of an abnormal NBS SCID result, since the initial lymphocyte subset quantitation revealed significant TCL, yet, HCT would not have been the correct treatment. For Cases 1 and 2, the presence of naïve T cells and partially preserved thymic function suggested an alternative diagnosis to SCID. Conversely, the overwhelming presence of memory T cells in Case 3 uncovered an aberrant T cell population, while also emphasizing the fact that absence of circulating naive T cells does not preclude an etiology extrinsic to the hematopoietic system. Case 4 underscores the importance of naïve and memory T cell assessment in hypomorphic RAG1 deficiency, which might have been missed with routine T, B, and NK cell analysis, and where the appropriate treatment was HCT.

In summary, our survey data, limited though it is by the number of respondents, indicates that there remains a need to harmonize follow-up testing protocols for an abnormal NBS SCID result and for ongoing discussions regarding optimal protocols for confirming an abnormal NBS SCID result in premature and low birth weight infants. The cases presented here illustrate the utility of including naïve and memory T cells analysis along with T, B, and NK cell flow cytometry quantitation during the initial confirmation of an abnormal NBS SCID result.

## Figures and Tables

**Table 1:** CD3 T cell counts and functional analysis in T cell lymphopenic conditions identified by NBS SCID ^1^.

Cause of T cell Lymphopenia	CD3 T Cell Count (Cells/µL)	PHA ^2^ Response	Comments
Typical SCID	* <300 or >300 in the context of maternal engraftment	* < 10% of normal	Considered an emergency and treated by HCT, enzyme or gene therapy
Leaky SCID or Omenn Syndrome	* 300–1500/µL with few naïve T cells	* 10–50% of normal	Due to hypomorphic defects in known SCID genes, no evidence of maternal engraftment. Treatment includes HCT, enzyme or gene therapy
Variant SCID/ Idiopathic T cell lymphopenia	300–1500/µL	Impaired	No known pathogenic variant. May or may not require HCT
Syndromes with T-cell lymphopenia	≤1500 cells/µL	May be impaired	e.g., DiGeorge syndrome, CHARGE ^3^ syndrome, Jacobsen syndrome, RAC2 ^4^ defects, DOCK8 ^5^ deficiency, ataxia telangiectasia. May require a thymus transplant (DGS) or HCT
Secondary T-cell lymphopenia	≤1500 cells/µL		e.g., Intestinal lymphangiectasia, anasarca, gastroschisis, third-spacing, gastrointestinal atresia, cardiac surgery with/without thymectomy, congenital heart defects, congenital infection with HIV, and neonatal leukemia.
Preterm infants	≤1500 cells/µL		T cell lymphopenia generally resolves with age

* Published diagnostic criteria.^1^ Newborn Screening for Severe Combined Immunodeficiency, ^2^ Phytohemagglutinin, ^3^ Coloboma, Heart defects, Choanal Atresia, growth Retardation, Genitourinary abnormalities, and Ear abnormalities. ^4^ Ras-related C3 botulinum toxin substrate 2. ^5^ Dedicator of cytokinesis 8.

**Table 2:** Survey questions administered to the CIS membership.

A. Which of these screening protocols does your laboratory use? TREC analysis by PCR (a) One-step (failed TREC screen proceeds to confirmatory testing and follow up) (b) Two-step (failed TREC screen is followed up by a second PCR analysis) (c) Other
B. Which of these phenotype and function assays does your laboratory use for confirmatory testing following a failed NBS SCID screen? Check all that apply. (a) TBNK (lymphocyte subsets) (b) CD45RA and CD45RO analysis for naïve and memory T cells (c) CD31 for recent thymic emigrants (d) (b) plus, CCR7 for naïve and memory T cell subsets (e) (b) plus CD62L (L-selectin) for naïve and memory T cell subsets (f) Additional markers (please specify) (g) Lymphocyte stimulation with PHA (specify method: tritium incorporation, CFSE dilution, EdU or BrdU) (h) Other
C. Does your laboratory follow a tiered testing approach? (a) TBNK as first tier followed by naïve and memory T cells as second tier (b) TBNK as first tier followed by naïve and memory T cells AND lymphocyte stimulation with PHA as second tier (c) TBNK AND naïve and memory T cells as first tier, followed by PHA stimulation as second tier (d) TBNK AND naïve and memory T cells AND PHA stimulation as first tier (e) Other
D. Premature infants: (a) Age and/or weight cut-off? __________(weight); __________(age) (b) Is confirmatory testing performed for a failed NBS SCID result? Yes/No (c) Other comments

**Table 3:** Case 1, lymphocyte quantitation.

Parameter	Result	Reference Range (Cells/µL)
CD45+ALC	0.76	1.93–7.46 × 10^3^ (<5 years)
CD3+T cells	344	1484–5327
CD4+T cells	248	733–3181
CD8+T cells	94	370–2555
CD19+B cells	252	370–2306
CD16/56+NK cells	159	43–526

**Table 4:** Case 2, lymphocyte subset analysis.

Parameter	Result	Reference Range (cells/µL)
CD45+ALC	734	1561–4630
CD3+T cells	494	1204–2889
CD4+T cells	322	506–1644
CD8+T cells	145	336–1296
CD19+B cells	44	215–1230
CD16/56+NK cells	150	102–827
T cell subset	Result	Percentage of CD4 or CD8 T cells
CD45RA+CD4+	42%	>50%
CD45RO+CD4+	23%	<15% in a neonate
CD45RA+CD8+	91%	>50%
CD45RO+CD8+	1%	<5% in a neonate
CD45RA+CD4+CCR7+	41%	97% of CD45RA+CD4+
CD8+CD45RA+CCR7	87%	97% of CD45RA+CD8+
CD4+CD45RA+CD31+	23%	55% of CD4+CD45RA+

**Table 5:** Case 3, Lymphocyte subset analysis.

Parameter	Result (Birth)	Result (7 Weeks Old)	Reference Range (Cells/µL) **
CD3+ T cells	14	1100	2500–5500
CD4+ T cells	ND *	800	1600–4000
CD8+ T cells	ND *	348	560–1700
CD19+ B cells	671	2530	300–2000
CD16/56+ NK cells	276	3084	170–1100

* ND, not determined, ** [23].

**Table 6:** Case 4, lymphocyte subset analysis including naïve and memory T cells.

Lymphocyte Subset	Result	Reference Range (Cells/µL)
CD3	949	2300–7000
CD4	575	1700–5300
CD8	359	400–1700
CD4+CD45RA+	312	41–1121
CD4+CD45RO+	185	153–582
CD19	89	600–1900
CD16+ and/or CD56+	811	200–1400
TCR	2.1%	1–10.3%

**Table 7:** Utility of measurement of CD4+CD45RA+ T cells in the diagnosis of SCID, Alberta, Canada cohort (2009–2018).

SCID Dx	Age at Dx	CD3	Ref Range *	CD4	Ref Range *	CD8	Ref Range *	Naïve CD4 ^#^	Ref Range *	B	Ref Range *	NK	Ref Range *
Naïve T cells useful for dx (*n* = 4) **													
Reticular dysgenesis/Omenn	5 months	1370	2200–9200	1230	1600–6500	170	300–3400	40	1600–6000	430	520–2300	130	97–1990
Cartilage hair hypoplasia/Omenn	11 months	1960	1600–6700	1800	1000–4600	140	400–2100	10	1100–4300	20	600–2700	50	200–1200
RAG2/Omenn	1 month	28410	1900–8400	13450	1500–6000	15190	300–2700	81	1300–5700	0	180–3500	1610	140–1900
Unknown/Omenn	3 months	1040	2200–9200	980	1600–6500	80	300–3400	10	1600–6000	710	520–2300	280	97–1990
Naïve T cells not required for dx (*n* = 11)													
X-linked SCID	Prenatal	0	1900–8400	0	1500–1600	0	300–2700	0	1300–5700	720	180–3500	90	140–1900
CD3 delta	3 months	0	2200–9200	0	1600–6500	0	300–3400	1	1600–6000	902	520–2300	198	97–1990
CD3 delta	5 months	45	2200–9200	0	1600–6500	0	300–3400	0	1600–6000	4050	520–2300	450	97–1990
CD3 delta	5 months	0	2200–9200	0	1600–6500	0	300–3400	0	1600–6000	1190	520–2300	70	97–1990
CD3 delta	6 months	0	1400–11500	0	1000–7000	0	200–5400	0	800–7600	3170	130–6300	1850	68–3900
CD3 delta	7 months	0	1400–11500	0	1000–7000	0	200–5400	0	800–7600	1210	130–6300	550	68–3900
RAG2	10 months	60	1600–6700	40	1000–4600	0	400–2100	10	1100–4300	180	600–2700	40	200–1200
ADA deficiency	2 months	0	1900–8400	0	1500–6000	0	300–2700	0	1300–5700	0	180–3500	0	140–1900
Reticular dysgenesis	Prenatal	0	1900–8400	0	1500–6000	0	300–2700	0	1300–5700	590	180–3500	420	140–1900
JAK3	7 months	20	1400–11500	0	1000–7000	10	200–5400	0	800–7600	920	130–6300	40	68–3900
Unknown	3 months	272	2200–9200	112	1600–6500	52	300–3400	0	1600–6000	96	520–2300	304	97–1900

* Reference range units: cells/µL, # Defined as CD3+CD4+CD45RA+. ** Maternal engraftment ruled out with sorted chimerisms.

## Abbreviations

SCID	Severe Combined Immunodeficiency
NBS	Newborn Screen
TCL	T-cell lymphopenia
TREC	T-cell receptor excision circle
RTEs	Recent Thymic Emigrants

## References

[1] Picard C., Gaspar H.B., Al-Herz W., Bousfiha A., Casanova J.-L., Chatila T.A., Crow Y.J., Cunningham-Rundles C., Etzioni A., Franco J.L. (2017). International Union of Immunological Societies: 2017 Primary Immunodeficiency Diseases Committee Report on Inborn Errors of Immunity. J. Clin. Immunol..

[2] Bousfiha A., Jeddane L., Picard C., Ailal F., Gaspar H.B., Al-Herz W., Chatila T.A., Crow Y.J., Cunningham-Rundles C., Etzioni A. (2017). The 2017 IUIS Phenotypic Classification for Primary Immunodeficiencies. J. Clin. Immunol..

[3] Routes J., Grossman W.J., Verbsky J.W., Laessig R.H., Hoffman G.L., Brokopp C.D., Baker M.W. (2009). Statewide Newborn Screening for Severe T-Cell Lymphopenia. Jama.

[4] Nourizadeh M., Shakerian L., Borte S., Fazlollahi M.R., Badalzadeh M., Houshmand M., Alizadeh Z., Dalili H., Rashidi-Nezhad A., Kazemnejad A. (2018). Newborn screening using TREC/KREC assay for severe T and B cell lymphopenia in Iran. Scand. J. Immunol..

[5] Audrain M.A.P., Thomas C., Mirallié S., Bourgeois N., Sébille V., Rabetrano H., Durand-Zaleski I., Boisson R., Persyn M., Pierres C. (2014). Evaluation of the T-cell receptor excision circle assay performances for severe combined immunodeficiency neonatal screening on Guthrie cards in a French single centre study. Clin. Immunol..

[6] McGhee S.A. (2012). Public health comes to immune deficiency. Blood.

[7] Van der Burg M., Mahlaoui N., Gaspar H.B., Pai S.-Y. (2019). Universal Newborn Screening for Severe Combined Immunodeficiency (SCID). Front. Pediatr..

[8] Borte S., Reichenbach J. (2015). Newborn Screening for Primary Immunodeficiencies: Focus on Severe Combined Immunodeficiency (SCID) and Other Severe T-Cell Lymphopenias. Int. J. Neonatal Screen..

[9] Mauracher A., Pagliarulo F., Faes L., Vavassori S., Güngör T., Bachmann L.M., Schmid J.P. (2017). Causes of low neonatal T-cell receptor excision circles: A systematic review. J. Allergy Clin. Immunol. Pr..

[10] Mallott J., Kwan A., Church J., Gonzalez-Espinosa D., Lorey F., Tang L.F., Sunderam U., Rana S., Srinivasan R., Brenner S.E. (2012). Newborn Screening for SCID Identifies Patients with Ataxia Telangiectasia. J. Clin. Immunol..

[11] Fronková E., Klocperk A., Svatoň M., Novakova M., Kotrová M., Kayserová J., Kalina T., Keslova P., Votava F., Vinohradska H. (2014). The TREC/KREC Assay for the Diagnosis and Monitoring of Patients with DiGeorge Syndrome. PLoS ONE.

[12] Kwan A., Church J.A., Cowan M.J., Agarwal R., Kapoor N., Kohn D.B., Lewis D.B., McGhee S.A., Moore T.B., Stiehm E.R. (2013). Newborn screening for severe combined immunodeficiency and T-cell lymphopenia in California: Results of the first 2 years. J. Allergy Clin. Immunol..

[13] Patrawala M., Kobrynski L. (2019). Nonsevere combined immunodeficiency T-cell lymphopenia identified through newborn screening. Curr. Opin. Allergy Clin. Immunol..

[14] Kuo C., Garcia-Lloret M.I., Slev P., Bohnsack J.F., Chen K. (2017). Profound T-cell lymphopenia associated with prenatal exposure to purine antagonists detected by TREC newborn screening. J. Allergy Clin. Immunol. Pract..

[15] Ward C.E., Baptist A.P., Urbina E.M., Khoury P., McCoy C.E., Dolan L.M., Daniels S., Kimball T.R. (2013). Challenges of Newborn Severe Combined Immunodeficiency Screening Among Premature Infants. Pediatrics.

[16] Azzari C., La Marca G., Resti M. (2011). Neonatal screening for severe combined immunodeficiency caused by an adenosine deaminase defect: A reliable and inexpensive method using tandem mass spectrometry. J. Allergy Clin. Immunol..

[17] Kuo C., Chase J., Lloret M.I.G., Stiehm E.R., Moore T., Aguilera M.J.M., Siles J.L., Church J.A. (2013). Newborn screening for severe combined immunodeficiency does not identify bare lymphocyte syndrome. J. Allergy Clin. Immunol..

[18] Fuleihan R.L. (2011). DOCK8 deficiency, T cell receptor excision circles and newborn screening. Clin. Immunol..

[19] Shearer W., Dunn E., Notarangelo L.D., Dvorak C.C., Puck J.M., Logan B.R., Griffith L.M., Kohn D.B., O’Reilly R.J., Fleisher T.A. (2013). Establishing diagnostic criteria for severe combined immunodeficiency disease (SCID), leaky SCID, and Omenn syndrome: The Primary Immune Deficiency Treatment Consortium experience. J. Allergy Clin. Immunol..

[20] CLSI (2013). Newborn Blood Spot Screening for Severe Combined Immunodeficiency by Measurement of T-Cell Receptor Excision Circles.

[21] Knutsen A.P., Baker M.W., Markert M.L. (2011). Interpreting low T-cell receptor excision circles in newborns with DiGeorge anomaly: Importance of assessing naive T-cell markers. J. Allergy Clin. Immunol..

[22] Seet C.S., He C., Bethune M.T., Li S., Chick B., Gschweng E.H., Zhu Y., Kim K., Kohn N.B., Baltimore D. (2017). Generation of mature T cells from human hematopoietic stem and progenitor cells in artificial thymic organoids. Nat. Methods.

[23] Shearer W., Rosenblatt H.M., Gelman R.S., Oyomopito R., Plaeger S., Stiehm E., Wara D.W., Douglas S.D., Luzuriaga K., McFarland E.J. (2003). Lymphocyte subsets in healthy children from birth through 18 years of age. J. Allergy Clin. Immunol..

[24] Chan K., Puck J.M. (2005). Development of population-based newborn screening for severe combined immunodeficiency. J. Allergy Clin. Immunol..

[25] Buckley R.H. (2010). Transplantation of hematopoietic stem cells in human severe combined immunodeficiency: Longterm outcomes. Immunol. Res..

[26] Chan A., Scalchunes C., Boyle M., Puck J.M. (2010). Early vs. delayed diagnosis of severe combined immunodeficiency: A family perspective survey. Clin. Immunol..

[27] Lee A.Y., Frith K., Schneider L., Ziegler J.B. (2017). Haematopoietic stem cell transplantation for severe combined immunodeficiency: Long-term health outcomes and patient perspectives. J. Paediatr. Child Health.

[28] Pai S.-Y., Logan B.R., Griffith L.M., Buckley R.H., Parrott R.E., Dvorak C.C., Kapoor N., Hanson I.C., Filipovich A.H., Jyonouchi S. (2014). Transplantation outcomes for severe combined immunodeficiency, 2000–2009. N. Engl. J. Med..

[29] Heimall J., Logan B.R., Cowan M.J., Notarangelo L.D., Griffith L.M., Puck J.M., Kohn N.B., Pulsipher M.A., Parikh S.H., Martinez C. (2017). Immune reconstitution and survival of 100 SCID patients post–hematopoietic cell transplant: A PIDTC natural history study. Blood.

[30] Puck J.M. (2012). Laboratory technology for population-based screening for severe combined immunodeficiency in neonates: The winner is T-cell receptor excision circles. J. Allergy Clin. Immunol..

[31] Van der Spek J., Groenwold R.H., Van der Burg M., Van Montfrans J. (2015). (Joris) TREC Based Newborn Screening for Severe Combined Immunodeficiency Disease: A Systematic Review. J. Clin. Immunol..

[32] Baker M.W., Laessig R.H., Katcher M.L., Routes J.M., Grossman W.J., Verbsky J., Kurtycz D.F., Brokopp C.D. (2010). Implementing routine testing for severe combined immunodeficiency within Wisconsin’s newborn screening program. Public Health Rep..

[33] Thompson J.G., Wilkey J.F., Baptiste J.C., Navas J.S., Pai S.Y., Pass K.A. (2010). Development of a high throughput multiplexed TREC qPCR assay with internal controls for detection of severe combined immunodeficiency in population-based newborn screening. Clin. Chem..

[34] Verbsky J.W., Baker M.W., Grossman W.J., Hintermeyer M., Dasu T., Bonacci B., Reddy S., Margolis D., Casper J., Gries M. (2011). Newborn Screening for Severe Combined Immunodeficiency; The Wisconsin Experience (2008–2011). J. Clin. Immunol..

[35] Baker M., Atkins A., Grossman W., Seroogy C., Lindstrom M., Brokopp C., Routes J. (2011). T-cell Receptor Excision Circles of Newborns Are Associated with Gestational Age: Data from Wisconsin Newborn Screening for Severe Combined Immunodeficiency. J. Allergy Clin. Immunol..

[36] De Felipe B., Olbrich P., Lucenas J.M., Delgado-Pecellín C., Pavón-Delgado A., Marquez J., Salamanca C., Soler-Palacín P., González-Granado L.I., Ferreras-Antolín L. (2015). Prospective neonatal screening for severe T- and B-lymphocyte deficiencies in Seville. Pediatr. Allergy Immunol..

[37] Amatuni G.S., Sciortino S., Currier R.J., Naides S.J., Church J.A., Puck J.M. (2019). Reference intervals for lymphocyte subsets in preterm and term neonates without immune defects. J. Allergy Clin. Immunol..

[38] Verbsky J.W., Thakar M., Routes J. (2012). The Wisconsin approach to newborn screening for severe combined immunodeficiency. J. Allergy Clin. Immunol..

[39] Vogel B.H., Bonagura V.R., Weinberg G.A., Ballow M., Isabelle J., DiAntonio L., Parker A., Young A., Cunningham-Rundles C., Fong C.-T. (2014). Newborn screening for SCID in New York State: Experience from the first two years. J. Clin. Immunol..

[40] Bosticardo M., Yamazaki Y., Cowan J., Giardino G., Corsino C., Scalia G., Prencipe R., Ruffner M., Hill D.A., Sakovich I. (2019). Heterozygous FOXN1 Variants Cause Low TRECs and Severe T Cell Lymphopenia, Revealing a Crucial Role of FOXN1 in Supporting Early Thymopoiesis. Am. J. Hum. Genet..

[41] Paganini I., Sestini R., Contini E., Giotti I., Gensini F., Marozza A., Barilaro A., Porfirio B., Papi L., Capone G.L. (2017). A novel PAX1 null homozygous mutation in autosomal recessive otofaciocervical syndrome associated with severe combined immunodeficiency. Clin. Genet..

[42] Abraham R.S. (2019). Assessment of Functional Immune Responses in Lymphocytes. Clinical Immunology.

